# The clinical outcomes of anterior minimally invasive inverted PHILOS plate fixation for distal humeral shaft fractures

**DOI:** 10.1186/s13018-025-05560-2

**Published:** 2025-02-19

**Authors:** Gang Fu, Shen’ao Wang, Weiqiang Wu, Fengfei Lin, Renbin Li

**Affiliations:** 1 Department of Orthopedic, Fuzhou Second General Hospital, Fuzhou, 350007 China; 2 The School of Clinical Medicine, Fujian Medical University, Fuzhou, China

**Keywords:** Distal humeral shaft fractures, Minimally invasive surgery, PHILOS plate, Iatrogenic radial nerve injury, Fracture healing

## Abstract

**Background:**

The unique anatomy and biomechanics of the distal humerus make distal humeral shaft fractures a significant challenge in orthopedic surgery. Conventional posterior surgical approaches are associated with risks such as iatrogenic radial nerve injury and increased soft tissue trauma.

**Objective:**

This study aimed to assess the clinical efficacy of an inverted PHILOS plate using an anterior minimally invasive percutaneous approach for treating distal humeral shaft fractures.

**Methods:**

We enrolled 32 patients with distal humeral shaft fractures. The surgical technique involved an anterior minimally invasive percutaneous application of an inverted PHILOS plate. Outcome measures included operative time, intraoperative blood loss, incision length, fracture healing time, and functional scores (Constant-Murley and Mayo Elbow Performance Scores). Postoperative complications, including nerve injury and nonunion, were documented.

**Results:**

The mean operative time was 1.69 ± 0.66 h, with a median blood loss of 50 ml (IQR: 50–100 ml). The mean incision length was 10.9 ± 1.78 cm, and the mean time to fracture healing was 11.2 ± 3.68 weeks. Functional outcomes were satisfactory, with a mean Constant shoulder score of 92.69 ± 6.6 and a mean Mayo Elbow Performance Score of 91.4 ± 8.04. No instances of iatrogenic nerve injury or nonunion were observed.

**Conclusion:**

The anterior minimally invasive percutaneous approach using an inverted PHILOS plate is an effective method for treating distal humeral shaft fractures. This technique minimizes soft tissue damage, reduces the risk of iatrogenic radial nerve injury, and ensures reliable fracture stabilization, thus offering a promising alternative to conventional posterior approaches.

**Supplementary Information:**

The online version contains supplementary material available at 10.1186/s13018-025-05560-2.

## Introduction

Fractures of the distal third of the humeral shaft, also referred to as distal humeral shaft fractures, account for approximately 7% of all humeral shaft fractures [1], 16% of humeral fractures, and 3% of total body fractures [2]. The distal humerus’s unique anatomy marks the transition from the cylindrical shape of the midshaft to the triangular shape, creating a biomechanical weak point that is prone to fractures. These fractures often result from direct or rotational trauma, with external forces typically concentrated on the anterolateral and posteromedial aspects of the distal humerus [3], at approximately 74–83% of the humerus’s length, with peak stress at around 79.8% [4]. Clinically, fractures are often spiral or oblique, sometimes with an associated wedge-shaped fragment on the medial side of the shaft. The distal humerus’s irregular anatomy challenges the selection of a standardized surgical approach.

Surgical treatment reduces the nonunion rate from 17.6% (conservative treatment) to 5.6%. Conservative treatment also risks complications such as skin pressure sores, rotational and angular deformities, and long-term limitations in shoulder and elbow function [5–8]. Open reduction and internal fixation (ORIF) is a common surgical approach, with various methods documented. However, the lack of a standardized anatomical plate complicates stable fixation, especially with reconstruction plates. The limited length of the distal fragment often means insufficient screw insertion for reliable stability, increasing the risk of fixation failure during rehabilitation [9, 10].

The posterior approach is often used for these fractures due to good exposure and the application of anatomically contoured lateral plates. However, it requires extensive soft tissue dissection and exposure of the radial nerve, risking iatrogenic injury, reported at 16–31.1% [5, 11, 12]. Extensive dissection can also disrupt blood supply, resulting in nonunion rates from 2–10% [13].

From January 2018 to May 2021, we used an inverted PHILOS plate fixation technique via an anterior minimally invasive approach to treat distal humeral shaft fractures. This technique overcomes limitations of reconstruction plates by allowing for sufficient distal screw fixation without extensive radial nerve exposure, reducing the risk of iatrogenic nerve injury. The results are promising and are presented in this retrospective study.

## Materials and methods

### Inclusion and exclusion criteria

For this study, the inclusion criteria encompassed patients who were 14 years of age or older, those presenting with fresh, closed fractures of the distal humeral shaft, and individuals without any pre-existing dysfunction of the shoulder or elbow on the affected side. The exclusion criteria included subjects with open fractures, patients exhibiting preoperative radial nerve injury, individuals with multiple fractures affecting the same limb, and patients with concomitant head injuries or other conditions that could impede postoperative rehabilitation. These criteria were meticulously applied to ensure a homogeneous study population, which would accurately reflect the treatment outcomes of the anterior minimally invasive inverted PHILOS plate fixation for distal humeral shaft fractures.

## General information

This study enrolled a total of 32 patients who fulfilled the inclusion criteria, comprising 18 males and 14 females. Ages spanned from 16 to 77 years with an average age of 35.8 ± 18.6 years. Fractures were categorized based on the AO classification: 27 were classified as Type A, 4 as Type B, and 1 as Type C. The study was approved by the Ethics Committee of the Second Hospital Affiliated to Xiamen University in Fuzhou (Approval No. 2024308). Written informed consent was obtained from all participants prior to study enrollment.

## Surgical technique

Patients were positioned supine with the affected shoulder elevated using a pad. Anesthesia was induced using a combination of general anesthesia and brachial plexus block. A distal anterior incision of the humerus was created, starting 1 cm superior to the cubital fossa’s center and extending proximally for about 4–5 cm. The biceps brachii muscle was retracted medially to expose the brachialis muscle, with careful attention paid to preserving the musculocutaneous nerve that traverses its surface. The brachialis muscle was then longitudinally incised at its mid-lateral aspect to reveal the distal humeral cortex. A periosteal elevator was utilized to develop a soft tissue tunnel along the bone, starting from the distal incision.

For plate fixation, a PHILOS plate was selected based on the fracture configuration. The plate’s length was determined such that the distal end would rest above the coronoid fossa, while the proximal end would not surpass the surgical neck of the humerus. The plate was introduced through a proximal incision, which was approximately 4–5 cm in length, made by dissecting along the interval between the deltoid and pectoralis major muscles, thereby exposing the humeral cortex. The plate’s proximal portion was positioned lateral to the pectoralis major’s insertion site, and the distal portion was placed superior to the coronoid fossa. A Kirschner wire was used for temporary distal fixation with the elbow flexed and the arm in a neutral position. Following appropriate traction, a proximal Kirschner wire was also inserted for temporary fixation. Intraoperative fluoroscopy confirmed the positioning of the plate and the fracture’s alignment. Upon confirmation of satisfactory alignment, 4–5 locking screws were inserted distally, with additional standard screws employed for securing any free bone fragments. Subsequently, four locking screws were sequentially inserted proximally. Fluoroscopy was utilized once more to verify the positioning of the plate and the adequacy of screw length. The surgical site was irrigated, and the subcutaneous tissues and skin were closed in a layered fashion.

### Postoperative care and follow-up

In the initial three days following surgery, patients were directed by a rehabilitation therapist in functional exercises. These included repeated fist clenches on the affected side and passive range-of-motion exercises for the shoulder and elbow joints, along with the application of ice packs to facilitate reduction of swelling. Prior to discharge, patients were instructed in a variety of functional exercises such as pendulum movements for the shoulder, wall climbing exercises, and active and passive flexion-extension movements for the elbow, as well as forearm rotation exercises. Passive exercises were emphasized during the first postoperative week. Subsequent to discharge, outpatient follow-up was conducted to provide further guidance on functional exercises.

### Outcome measures

The study documented the operative duration, intraoperative blood loss, total length of the incision, and time to fracture healing. Patients were followed up at 1, 3, and 6 months post-surgery, with assessments of the Constant-Murley shoulder function score, the Mayo Elbow Performance Score, and the Visual Analog Scale (VAS) pain scores for both the shoulder and elbow. Any postoperative complications were meticulously recorded, including nerve injury, incision infection, and fracture nonunion.

### Statistical analysis

Data were analyzed using IBM SPSS Statistics software, version 26.0. The normality of the data distribution for continuous variables was assessed with the Shapiro-Wilk test. Variables including follow-up duration, age, length of the distal fracture, count of distal screws, operative time, total incision length, time to fracture healing, Constant-Murley scores, and Mayo Elbow Performance scores were normally distributed and are expressed as mean ± standard deviation (x̄ ± s). Non-normally distributed variables, including blood loss, shoulder Visual Analog Scale (VAS) scores, and elbow VAS scores, are reported as median (M).

## Results

All patients completed the follow-up. The mean age of the patients was 35.8 ± 18.6 years (range, 16–77 years). The mean operative time was 1.69 ± 0.66 h (range, 1–3.5 h). The median intraoperative blood loss was 50 ml (IQR: 50–100 ml). The total incision length averaged 10.9 ± 1.78 cm (range, 7–16 cm). The follow-up duration was 14.19 ± 7.44 months (range, 10–30 months) (Table 1). The mean time to fracture healing was 11.2 ± 3.68 weeks (range, 6–18 weeks).The mean Constant shoulder function score was 92.69 ± 6.6 (range, 80–100), and the mean Mayo Elbow Performance Score (MEPS) was 91.4 ± 8.04 (range, 75–100). The median shoulder VAS score was 1 (IQR: 0, 2), and the median elbow VAS score was 1 (IQR: 0, 1.75) (Table 2). The mean distal cortical length of the fracture was 3.93 ± 1.75 cm (range, 2.13–10.9 cm). A mean of 5.72 ± 1.33 screws (range, 3–9) were used for fixation in the distal fragment (Table 3, Figs. A and B).

**Fig. A:**
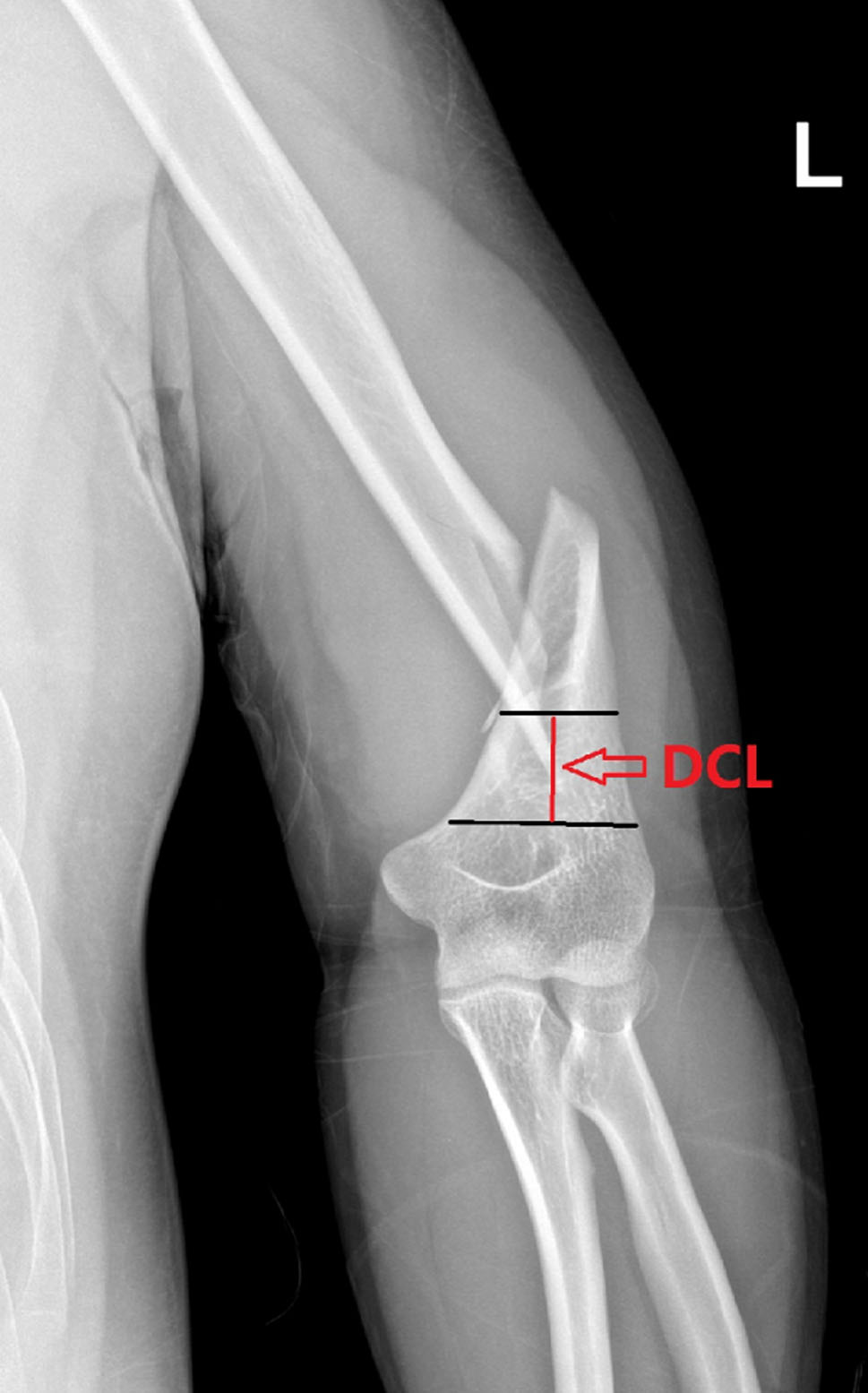
The distal cortical length (DCL) is defined as the distance from the upper edge of the olecranon to the distal end of the fracture line. Our study, based on the schematic diagram for PHILOS plate length measurement, revealed that for PHILOS plates, a DCL of 2.5 cm is required for 6 screws, 3 cm for 7 screws, and 4 cm for 9 screws. Compared to other types of plates, the PHILOS plate can accommodate a greater number of screws within the same DCL, thereby enhancing the strength of fracture fixation and maintaining stability. This design advantage potentially offers clinical benefits in providing more reliable fracture stabilization

**Fig. B:**
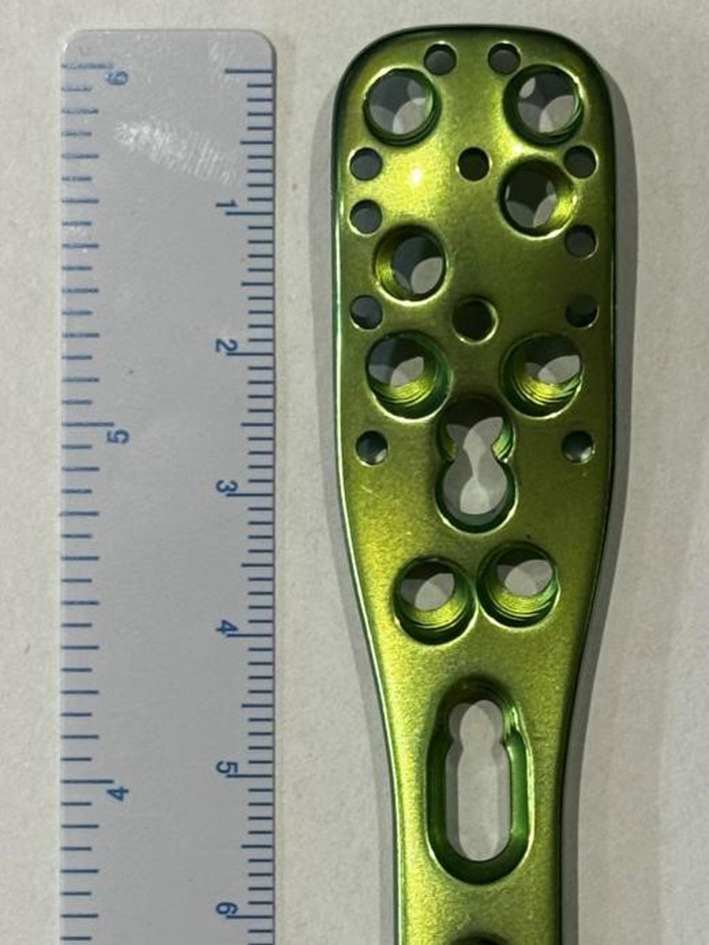
Schematic diagram of PHILOS plate length measurement

**Table 1:** Characteristics of patients with distal third Diaphyseal fractures of the Humerus

Characteristics	Values (*N* = 32 patients)
Mean age (years ± SD)	35.81 ± 18.59
Sex	
Male, n (%)	18(56.3%)
Female, n (%)	14(43.7%)
Side	
Right humerus injury, n (%)	21(65.6%)
Left humerus injury, n (%)	11(34.4%)
Injury mechanism, n (%)	
Motor vehicle accident injury	15(46.9%)
Fall down	10(31.2%)
Arm wrestling	7(21.9%)
AO classification, n (%)	
A1	25(78.1%)
A3	2(6.3%)
B1	4(12.5%)
C1	1(3.1%)
Mean follow-up, n (months ± SD)	14.19 ± 7.44

**Table 2:** Operation related and follow-up data

Characteristics	Values
Surgery time (hour)	1.69 ± 0.66
Blood loss (mL)	50(50, 100)
Total incision length (mm)	10.90 ± 1.78
Healing time (Weeks)	11.2 ± 3.68
Constant score	92.69 ± 6.60
MEPS score	91.43 ± 8.04
VAS (Shoulder)	1(0, 2)
VAS (Elbow)	1(0, 1.75)

Note: Constant score of shoulder in the last follow-up (Constant), Mayo Elbow Performance Score, MEPS, Visual Analogue Scale for Shoulder Pain and Visual Analogue Scale for Elbow Pain

**Table 3:** The relationship between the proximal length of PHILOS plate and the number of screws, the length of distal fracture block of lower humeral shaft fracture and the number of screws inserted

Characteristics	Values
Proximal Length of Plate and Corresponding Screw Holes	
2.5 c*m*:	6 holes
3.0 cm:	7 holes
4.0 cm:	9 holes
Distal cortical length (DCL) (cm ± SD)	3.93 ± 1.75
Distal screws (n ± SD)	5.72 ± 1.33

## Discussion

In this study, we adopted an anterior minimally invasive percutaneous approach using an inverted PHILOS plate for the fixation of distal humeral shaft fractures. This method was designed to enhance distal fragment fixation by allowing for an increased number of screws while minimizing damage to the surrounding soft tissues. Multiple small incisions (average total length: 10.9 cm) minimized soft tissue injury and intraoperative blood loss, with a mean volume of only 50 mL. The minimally invasive surgical technique, coupled with indirect fracture reduction, maximized the preservation of the blood supply to the fracture site, which we believe significantly contributed to the absence of nonunion cases observed in our study. Furthermore, the approach eliminated the risk of iatrogenic nerve injury, wound infection, and other complications, demonstrating the safety and effectiveness of this technique in managing distal third diaphyseal fractures of the humerus.

A posterior approach utilizing a laterally positioned anatomical plate on the distal humerus is a prevalent surgical technique for treating distal humeral shaft fractures [14, 15]. However, this method necessitates proximal dissection and exposure of the radial nerve, which is associated with a risk of iatrogenic nerve injury due to the repeated traction involved in the surgical procedure [16]. Literature reports indicate that the incidence of such nerve injuries can range from 16–31.1% [5, 11, 12]. Moreover, the extensive soft tissue dissection characteristic of the posterior approach may disrupt the blood supply to the fracture site, thereby increasing the risk of nonunion [17–19].

Conversely, recent studies have investigated the application of minimally invasive anterior single-plate fixation for midshaft humeral fractures [18]. This technique offers several advantages, such as reduced incision size, minimal soft tissue damage, and the avoidance of radial nerve exposure, which collectively contribute to a decreased risk of iatrogenic nerve injury and enhanced fracture healing rates [20, 21]. Nonetheless, when addressing distal third diaphyseal fractures, the limited length of the distal fragment and the irregular morphology of the distal humerus pose significant challenges. Currently, there is an absence of anatomical plates specifically contoured for this region. Utilizing a reconstruction plate in such cases may only permit the insertion of 2 or 3 screws into the distal fragment, which may not provide adequate fixation strength, thereby raising the risk of implant failure during the early stages of functional rehabilitation [9, 22–24].

In our study, the average cortical length of the distal fragment was measured to be 3.93 cm. Our findings revealed that for PHILOS plates, a plate length of 2.5 cm was required for proximal fixation with 6 screws, 3.0 cm for 7 screws, and 4.0 cm for 9 screws. We measured the length of the bone cortex available for screw insertion at the distal end of the fracture (DCL), and the results showed an average length of 5.72 ± 1.33 cm.With an average of 5.7 screws inserted into the distal fragment in our series, this approach ensured a sufficient number of screws for optimal fracture stability compared to the use of reconstruction plates, highlighting the importance of selecting appropriate plate lengths and screw configurations for effective fracture management and early functional recovery.

**Fig. 1:**
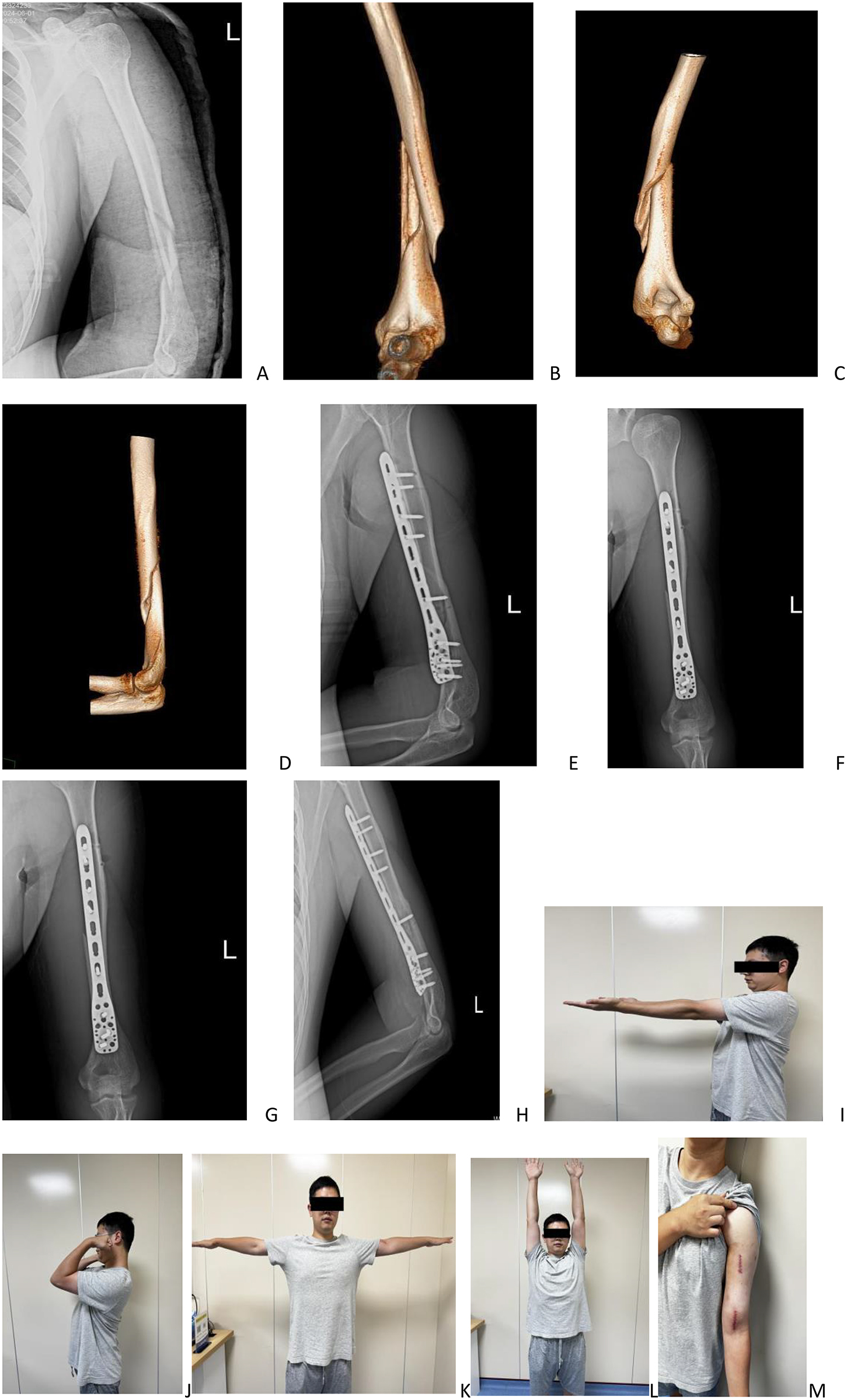
(Patient A): a 33-year-old male, presented with pain in his left upper arm following a fall. Radiographic evaluation, including X-ray and CT imaging, confirmed a fracture involving the distal segments of the left humerus **(A-D)**. The fracture was treated using an inverted PHILOS plate fixation via an anterior minimally invasive surgical approach **(E-F)**. A follow-up X-ray taken two months postoperatively demonstrated satisfactory fracture healing **(G-H)**. At follow-up, the patient’s left elbow exhibited a functional range of motion from 0° to 130°. The patient achieved full functional recovery, as reflected by a score of 100 on both the Mayo Elbow Performance Score (MEPS) and the Constant-Murley shoulder score **(I-L)**. The total length of the surgical incisions was approximately 15 cm, and the incisions healed without complications **(M)**

**Fig. 2:**
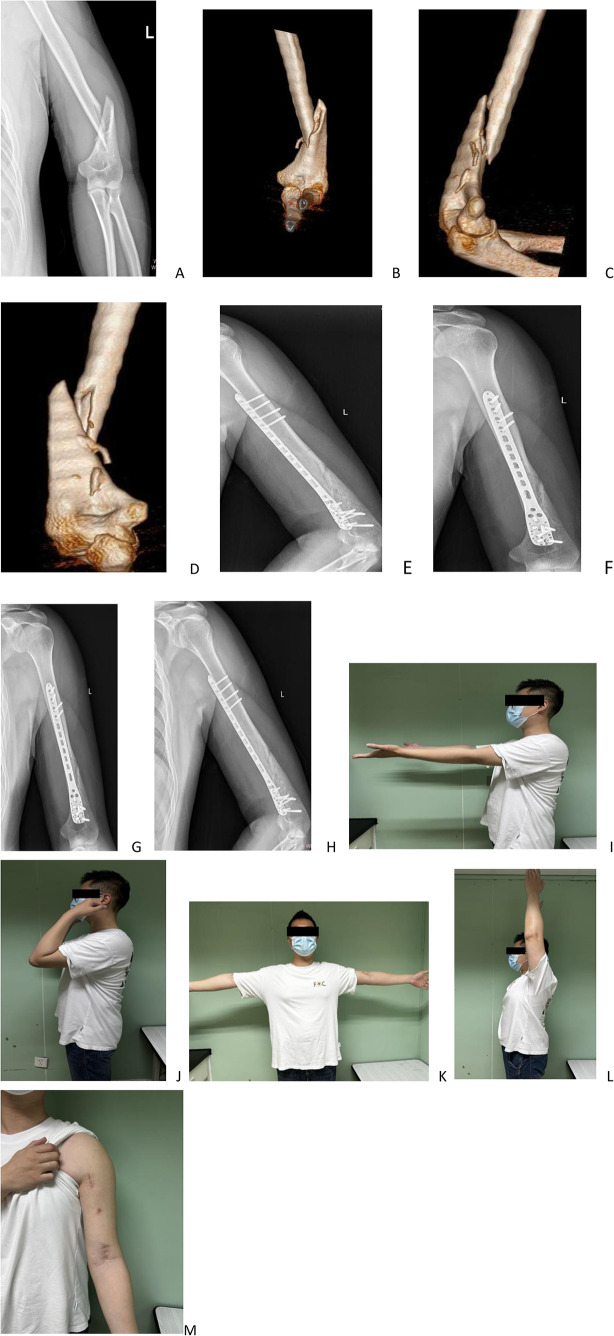
(Patient B): a 31-year-old male, presented with pain in his left upper arm following a fall. Radiographic evaluation, including X-ray and CT imaging, confirmed a spiral fracture of the humerus **(A-D)**. The fracture was treated using an inverted PHILOS plate fixation via an anterior minimally invasive surgical approach **(E-F)**. A follow-up X-ray taken two months postoperatively demonstrated satisfactory fracture alignment and healing progress **(G-H)**. At follow-up, the patient’s left elbow exhibited a functional range of motion from 0° to 135°. The patient achieved a Mayo Elbow Performance Score (MEPS) of 95 and a Constant-Murley shoulder score of 98, reflecting a satisfactory functional recovery **(I-L)**. The total length of the surgical incisions was approximately 13 cm, and the incisions healed without complications **(M)**

Iatrogenic radial nerve injury is a well-documented complication associated with open reduction and internal fixation (ORIF) via the posterior approach for distal humeral shaft fractures [25, 26]. Jawa et al. [5] reported three cases of iatrogenic radial nerve injury among 19 patients who underwent posterior approach fixation; these patients began to show signs of nerve recovery at three months postoperatively, with complete recovery by six months. Prasarn et al. [27] also documented three cases of iatrogenic radial nerve injury in a series of 15 patients treated with dual-plate fixation via the posterior approach, with full recovery occurring between five to seven months postoperatively. Generally, nerve palsy symptoms resulting from intraoperative traction on the radial nerve are known to resolve within 4 to 6 months, often without the need for immediate surgical exploration [28]. However, this recovery period can lead to increased psychological and financial burdens on patients and may prolong the functional recovery of the upper limb.

Studies have indicated that the posterior approach carries a higher risk of iatrogenic radial nerve injury compared to anterior minimally invasive plate fixation in the treatment of distal humeral shaft fractures [29–31]. The risk of radial nerve injury is further elevated in cases requiring revision surgery for nonunion or secondary implant removal [5, 16]. The anterior minimally invasive plating technique, which does not require exposure of the radial nerve during surgery, has been shown to reduce the risk of nerve injury. In our study, none of the patients exhibited postoperative nerve injury symptoms, underscoring the significant advantage of this technique in avoiding iatrogenic radial nerve injury and supporting its use in clinical practice for the treatment of distal humeral shaft fractures (Typical cases: A and B).

## Limitation

This study acknowledges several limitations that merit consideration: (1) The sample size was relatively small, which may limit the generalizability of our findings. (2) As the anterior approach does not allow direct visualization of the radial nerve, it may not be suitable for cases requiring nerve exploration, such as those with preoperative radial nerve injury. (3) The technique, which involves minimally invasive procedures and closed reduction, has an associated learning curve that surgeons must overcome to master this approach. (4) Further biomechanical studies are necessary to validate the effectiveness of this surgical method, ensuring that it provides the required stability and supports optimal functional outcomes.

## Conclusion

In summary, the use of an inverted PHILOS plate with an anterior minimally invasive percutaneous approach for the treatment of distal humeral shaft fractures provides several benefits. These include minimal soft tissue damage, reduced intraoperative blood loss, and a lower risk of iatrogenic radial nerve injury. This technique proves to be an effective method for the clinical management of such fractures.

## Electronic supplementary material

Below is the link to the electronic supplementary material.

Supplementary Material 1

## Data Availability

No datasets were generated or analysed during the current study.

## References

[1] Rockwood CA, Green DP, Bucholz RW. Rockwood and Green’s fractures in adults, 8th ed[M]. Volume 1. Philadelphia: Lippincott-Raven; 2015. p. 1230.

[2] Ekholm R, Adami J, Tidermark J, et al. Fractures of the shaft of the humerus. An epidemiological study of 401 fractures. J Bone Joint Surg Br. 2006;88(11):1469–73. 10.1302/0301-620X.88B11.17634. 17075092 10.1302/0301-620X.88B11.17634

[3] Sakai K, Kiriyama Y, Kimura H, et al. Computer simulation of humeral shaft fracture in throwing. J Shoulder Elb Surg. 2010;19(1):86–90. 10.1016/j.jse.2009.05.006. 10.1016/j.jse.2009.05.006 19574067

[4] Duan MS, Cao K, Wang JH., et al.Morphology and clinical significance of the distal intramedullary bony crista of the humerus. Chin J Clin Anat 2008, 26(5):500–2. 10.3969/j.issn.1001-165X.2008.05.010.

[5] Jawa A, Mccarty P, Doornberg J, et al. Extra-articular distal-third diaphyseal fractures of the humerus. A comparison of functional bracing and plate fixation. J Bone Joint Surg Am. 2006;88(11):2343–7. 10.2106/JBJS.F.00334. 17079389 10.2106/JBJS.F.00334

[6] Harris IA, Mourad M, Kadir A et al. Publication bias in abstracts presented to the annual meeting of the American Academy of Orthopaedic Surgeons. J Orthop Surg (Hong Kong), 2007, 15(1): 62–66. 10.1177/230949900701500114 10.1177/230949900701500114 17429120

[7] Sargeant HW, Farrow L, Barker S, et al. Operative versus non-operative treatment of humeral shaft fractures: a systematic review. Shoulder Elb. 2020;12(4):229–42. 10.1177/1758573218825477. 10.1177/1758573218825477 PMC7400715 32788928

[8] Sarmiento A, Horowitch A, Aboulafia A, et al. Functional bracing for comminuted extra-articular fractures of the distal third of the humerus. J Bone Joint Surg Br. 1990;72(2):283–7. 10.1302/0301-620X.72B2.2312570. 2312570 10.1302/0301-620X.72B2.2312570

[9] Westrick E, Hamilton B, Ioogood P, et al. nt Orthop. 2017;41(2):385–95. 10.1007/s00264-016-3210-7. 10.1007/s00264-016-3210-7 27150488

[10] Wang LJ, Shi YX, Shao WZ, et al. Osteosynthesis of mid-distal humeral diaphyseal fracture with an anatomically precontoured extra-articular distal plate system. Chin J Orthop Trauma. 2017;19(10):907–10. 10.3760/cma.j.issn.1671-7600.2017.10.014.

[11] Dutta A, Kotoky AJ, Sipani AK, et al. Clinical study on the management of extra-articular distal humerus fracture treated with extra-articular distal humeral locking compression plate. Int J Orthop Sci. 2019;5(1):1–6. 10.22271/ortho.2019.v5.i1a.01.

[12] An Z, Zeng B, He X, et al. Plating osteosynthesis of mid-distal humeral shaft fractures: minimally invasive versus conventional open reduction technique. Int Orthop. 2010;34(1):131–5. 10.1007/s00264-009-0753-x. 19301000 10.1007/s00264-009-0753-x PMC2899279

[13] Kashayi-Chowdojirao S, Vallurupalli A, Chilakamarri VK, et al. Role of autologous non-vascularised intramedullary fibular strut graft in humeral shaft nonunions following failed plating. J Clin Orthop Trauma. 2017;8(Suppl 2):S21–30. 10.1016/j.jcot.2016.12.006. 29339841 10.1016/j.jcot.2016.12.006 PMC5761704

[14] Lee JK, Choi YS, Sim YS, et al. Dual plate fixation on distal third diaphyseal fracture of the humerus. Int Orthop. 2017;41(8):1655–61. 10.1007/s00264-016-3355-4. 27909754 10.1007/s00264-016-3355-4

[15] Zhang X, Yang J, Wang W, et al. A short-term clinical efficacy of dual plate vs.single plate in treatment of AO type C mid-distal humeral fractures. Chin J Bone Joint Injury. 2018;33(09):35–8. 10.7531/j.issn.1672-9935.2018.09.009.

[16] Gallucci GL, Boretto JG, Alfie VA, et al. Posterior minimally invasive plate osteosynthesis(MIPO)of distal third humeral shaft fractures with segmental isolation of the radial nerve. Chir Main. 2015;34(5):221–6. 10.1016/j.main.2015.06.007. 26388162 10.1016/j.main.2015.06.007

[17] Xue Z, Ding H, Hu C, et al. An anatomical study of the nutrient Foramina of the human humeral Diaphysis. Med Sci Monit. 2016;22:1637–45. 10.12659/MSM.898361. 27180828 10.12659/MSM.898361 PMC4917311

[18] Apivatthakakul T, Arpornchayanon O, Bavornratanavech S. Minimally invasive plate osteosynthesis(MIPO) of the humeral shaft fracture. Is it possible? A cadaveric study and preliminary report. Injury. 2005;36(4):530–8. 10.1016/j.injury.2004.05.036. 15755436 10.1016/j.injury.2004.05.036

[19] Xue ZC, Jiang CL, Qin H, et al. Influence of minimal invasive anterior plating osteosynthesis on blood supply to the middle and distal third humeral shaft. Chin J Orthop Trauma. 2014;16(10):881–4. 10.3760/cma.j.issn.1671-7600.2014.10.012.

[20] Pidhorz L. Acute and chronic humeral shaft fractures in adults. Orthop Traumatol Surg Res. 2015;101(Supplement):S41–9. 10.1016/j.otsr.2014.07.034. 25604002 10.1016/j.otsr.2014.07.034

[21] Kevin T. Minimally invasive plate osteosynthesis of humeral shaft fractures: current state of the art. J Am Acad Orthop Surg. 2018;15(18):652–61. 10.5435/JAAOS-D-17-00238. 10.5435/JAAOS-D-17-00238 30113346

[22] Scolaro JA, Hsu JE, Svach DJ, et al. Plate selection for fixation of extra-articular distal humerus fractures: a biomechanical comparison of three different implants. Injury. 2014;45(12):2040–4. 10.1016/j.injury.2014.08.036. 25249244 10.1016/j.injury.2014.08.036

[23] Gao F, Wang XH, Xia SL, et al. Single and dual-plate internal fixation via posterior approach in the treatment of distal-third humeral shaft fracture. Chin J Bone Joint. 2018;7(11):21–4. 10.3969/j.issn.2095-252X.2018.11.004.

[24] Rajesh V, Chawda, Vijay J, et al. A distal third extra-articular humerus fractures treated with precontoured single anatomical locking plates: a retrospective study of 11 cases. Nat J Clin Orthop. 2019;3(1):22–5. 10.33545/orthor.2019.v3.i1a.07.

[25] Feng Q, He X, Sun L, et al. Progress in diagnosis and treatment of fracture-related infection. Int J Surg. 2020;47(11):782–7. 10.3760/cma.j.issn115396-20200820-00255.

[26] Ilyas AM, Mangan JJ, Graham J. Radial nerve Palsy Recovery with fractures of the Humerus: an updated systematic Review. J Am Acad Orthop Surg. 2020;28(6):e263–9. 10.5435/JAAOS-D-18-00142. 31714418 10.5435/JAAOS-D-18-00142

[27] Prasarn ML, Ahn J, Paul O, et al. Dual plating for fractures of the distal third of the humeral shaft. J Orthop Trauma. 2011;25(1):57–63. 10.1097/BOT.0b013e3181df96a7. 21085023 10.1097/BOT.0b013e3181df96a7

[28] Ljungquist KL, Martineau P, Allan C. Radial nerve Injuries. J Hand Surg Am. 2015;40(1):166–72. 10.1016/j.jhsa.2014.05.010. 25442768 10.1016/j.jhsa.2014.05.010

[29] Schwab TR, Stillhard PF, Schibli S, et al. Radial nerve palsy in humeral shaft fractures with internal fixation: analysis of management and outcome. Eur J Trauma Emerg Surg. 2018;44(2):235–43. 10.1007/s00068-017-0775-9. 28280873 10.1007/s00068-017-0775-9 PMC5884898

[30] Chen TY, Han PF, Li PC, et al. Minimally invasive plate osteosynthesis versus open reduction and internal fixation for humeral shaft fractures: a Meta-analysis. Chin J Orthop Trauma. 2019;021(005):416–21. 10.3760/cma.j.issn.1671-7600.2019.05.010.

[31] Apivatthakakul T, Patiyasikan S, Luevitoonvechkit S. Danger zone for locking screw placement in minimally invasive plate osteosynthesis(MIPO) of humeral shaft fractures: a cadaveric study. Injury. 2010;41(2):169–72. 10.1016/j.injury.2009.08.002. 19735916 10.1016/j.injury.2009.08.002

